# Effects of 6-month, Multimodal Exercise Program on Clinical and Gait Parameters of Patients with Idiopathic Parkinson's Disease: A Pilot Study

**DOI:** 10.5402/2011/714947

**Published:** 2011-10-30

**Authors:** Rodrigo Vitório, Claudia Teixeira-Arroyo, Ellen Lirani-Silva, Fabio Augusto Barbieri, Maria Joana Duarte Caetano, Sebastião Gobbi, Florindo Stella, Lilian Teresa Bucken Gobbi

**Affiliations:** ^1^UNESP-São Paulo State University, Avenue 24-A-1515, 13506-900 Rio Claro, SP, Brazil; ^2^Gait and Posture Studies Laboratory, Physical Education Department-UNESP, Avenue 24-A-1515, 13506-900 Rio Claro, SP, Brazil; ^3^UNICAMP-Campinas State University, SP, Brazil

## Abstract

This pilot study aimed to identify the effects of a 6-month, multimodal exercise program on clinical and gait parameters in patients with Parkinson's disease. Two groups of participants were enrolled in the study: Trained Group (TG) and Control Group (CG). Individuals in the TG exercised three times a week for 24 weeks (in a multimodal exercise program), while the CG participants maintained their regular activity level. Participants in both the TG and the CG were assessed at pre- and posttest by clinical rates and the spatiotemporal parameters of self-paced walking. The two groups were not significantly different in demographic, clinical, and gait data at baseline. There were no significant differences between groups for clinical data at posttest. The purposed multimodal exercise program has found improvement in some kinematic gait parameters for the TG. Further study in the form of randomized controlled trial would be required to establish effectiveness of the intervention.

## 1. Introduction

Parkinson's disease (PD) affects approximately 0.3% of the population worldwide, and from 1% to 2% of individuals are more than 60 years old [1]. In Brazil, a recent population-based cohort study showed PD to have a prevalence of 3.3% [2]. PD is a neurodegenerative pathology characterized by progressive degeneration of the dopamine-containing neurons in the *substantia nigra pars compacta*. The decreased amount of dopamine compromises the optimum amount of neuromotor impulses required for the accurate control of muscle activation. As a consequence, PD patients show motor disturbances (e.g., resting tremor, rigidity, postural instability, and gait disorders). The clinical parameters of PD patients tend to get worse progressively [3], even though therapeutic interventions have shown some benefits to patients [4, 5]. 

Gait disorders are one of the most incapacitating signs of PD. The negative impact of gait disorders includes immobility (causing loss of independence) and the risk of falling. Therefore, a large number of studies have been performed to measure gait parameters in PD patients. These studies have shown that Parkinsonian gait is characterized by shortened step and stride length and reduced velocity [6, 7]. While cadence typically is not modified, in some cases, as a possible adaptation to amplitude regulation disorder, it appears to increase [6]. These gait features progressively worsen with the advance of the disease, which severely limits patients' mobility and quality of life [8, 9].

Researchers have examined empirical studies to establish effective interventions that can improve the gait parameters of PD patients. For example, a recent meta-analysis revealed that exercise trials may promote benefits related to gait in PD patients [10]. Some studies achieved satisfactory results by applying specific exercise programs for locomotion (e.g., body-weight-supported exercises on a treadmill [11, 12], walking on a treadmill at a speed greater than overground walking speed [13], use of visual and auditory cues [14]), for lower limb strength [15] and for coordination and sensory attention [4, 5]. Thus, we can speculate that specificity of exercise is not a critical factor in improving gait parameters of PD patients.

Although promising, studies of exercise in PD have been limited in scope (program duration and specificity). Most have addressed the effects of short-term (typically implemented over 4 to 12 weeks) specific exercise programs. The benefits of longer and nonspecific exercise intervention remain poorly understood [16, 17]. To our knowledge, this is the first study addressing the effects of a 6-month, multimodal exercise program on gait parameters in PD patients. Our research group has previously demonstrated a positive effect of the purposed program on executive functions [16] and balance [17].

Within this context, the current study employed a broader approach, one which utilizes a multimodal exercise program, in an attempt to improve the gait parameters of PD patients. In addition, many exercise trial studies have ignored the overall changes in symptoms or disease severity [18, 19]. Sage and Almeida [4] suggested that in exercise trials it is crucial for investigators to consider effects on clinical symptoms in conjunction with changes in gait. Therefore, the aim of this study was to identify the effects of a 6-month, multimodal exercise program on clinical and kinematic gait parameters in PD patients. We expected to observe positive changes in PD patients' symptoms and gait parameters after their participation in the program.

## 2. Methods

This study adhered to the guidelines of the Declaration of Helsinki and was approved by the local Ethics Committee. All patients signed a consent form before involvement in the study. 

### 2.1. Participants

The participants were recruited through the assistance of physicians (neurologists and psychiatrists) from Rio Claro, São Paulo, Brazil, who encouraged their patients to participate in the study. Thirty-four patients with PD volunteered to participate in the study. All had a diagnosis of idiopathic PD, with no other major neurological problems. Diagnosis of PD was made according to the United Kingdom Parkinson's disease Society Brain Bank clinical criteria for idiopathic PD. The participants were assigned to two groups according to personal interest (the participants chose which group they preferred to be part of): Trained Group (TG; *n* = 24) and Control Group (CG; *n* = 10). Participants of both groups were sedentary prior to the study (subjects had followed any training program during last year). Individuals in the TG participated in a 6-month, multimodal exercise program described under the training protocol section. Participants in the CG kept to their same daily routines and did not participate in any regular or structured exercise during the study period.  Table 1 shows demographic data at baseline for the 29 individuals who completed the study. Levodopa intake remained unchanged for all participants during the intervention. Inclusion criteria were disease in Stages I–III of the Hoehn and Yahr Rating Scale (H&Y) [20], independent walker, and no cognitive impairment, as judged by the Mini-Exam of Mental Status (MEMS) [21]. Brucki et al.'s [22] suggestions for utilization of the MEMS in Brazil (cutoff score according to educational level) were followed to screen for cognitive impairment. Exclusion criteria were any history of orthopedic, cardiovascular, or psychiatric disorders, as judged by the clinical assessment. No participant suffered from freezing of gait.

### 2.2. Training Protocol

The aim of the multimodal exercise program was to develop the patients' functional capacities, cognitive functions, posture, and locomotion. In contrast to specific programs, this one targeted a global (holistic) improvement of PD patients. It is composed of a variety of activities that simultaneously focus on other components of functional capacity, such as muscular resistance (specific exercises for gastrocnemius, quadriceps femoralis, hamstring, rectus abdominalis, and trunk dorsal muscles), motor coordination (rhythmic activities), and balance (recreational motor activities). These components were selected because they seem to be affected by PD and could represent underlying mechanisms to gait impairments. The multimodal program took place over a 6-month period (72 sessions, 3 times a week, and 60 minutes per session). Each session consisted of five parts (warm-up, preexercise stretching, exercise session, cool down and postexercise stretching). The main exercise session lasted 40 minutes. The program was divided into six phases; each phase was composed of 12 sessions, each lasting approximately one month. At the end of each phase there was a progressive increase of load (Table 2). In each session, three different participants utilized a heart rate monitor (Polar) to assess the intensity of the session. Heart rate during the main exercise sessions remained between 60% and 80% of maximum heart rate (220 minus the participant's age in years).

Each participant was required to participate in at least 70% of the sessions in order to be included in the data analysis. No participant of the TG was absent from the intervention program for more than five consecutive sessions. The exercise program was supervised by at least three physical education professionals each time. No adverse events with the intervention were perceived or reported.

### 2.3. Evaluation

Participants were tested before commencing the multimodal program (pretest) and upon completion (posttest). All assessments were carried out in the morning, in the “on-medication” state, 1 hour after participants' first morning dose of medication.

A neuropsychiatrist performed a clinical assessment in order to test participants on the Unified Parkinson's Disease Rating Scale (UPDRS) [23], MEMS, and H&Y. Higher scores on the UPDRS signify higher deficit levels of the disease. Conversely, higher scores on the MEMS represent a more preserved cognitive function. For data analysis, scores on the UPDRS subsections I (Mentation, Behavior, and Mood), II (Activities of Daily Living), and III (Motor) were considered separately. The rater was blinded as to the study purpose and to the groups in which the patients participated.

The walking task required participants to walk, at a self-paced speed, on a pathway 8 m long by 1.4 m wide, which was covered with a black rubber carpet, 3 mm thick. Three trials were performed. For the kinematic data recording, two passive markers (reflective, adhesive Styrofoam, 15 mm in diameter) were attached to the following anatomic landmarks: lateral face of the right calcaneus and medial face of the left calcaneus. The images of the right sagittal plane of one stride at center of the pathway were recorded with a frequency of 60 Hz by one digital camcorder (JVC, GR-DVL 9800), generating 2D kinematic data. Markers were digitized automatically on Digital Video for Windows (DVIDEOW) software [24]. The x and y coordinates for each marker were transformed into a metric system by means of a bidimensional reference system, with four control points, and with a length of 1478 mm and a height of 1480 mm. An experiment error of 4.61 mm was obtained. Raw data were filtered using a low-pass, second-order digital Butterworth filter, with a cutoff frequency of 5 Hz in the Matlab 6.5 environment. A Matlab algorithm calculated the gait-dependent variables by manipulating the matrix created by the DVIDEOW software.

The following gait-dependent variables were calculated on the central right stride, from heel contact to the next heel contact: stride length, stride duration, stride velocity, cadence, double-support phase duration, single-support phase duration, and swing phase duration [25, 26]. The values for each dependent variable of all three trials per participant were considered for statistical analysis. This particular set of gait parameters was chosen because it has been shown to be altered in PD and to be a sensitive tool to identify changes in gait after the enrolment in exercise programs [4, 6, 13, 25, 26]. The personnel conducting gait analyses were also blinded as to the study purpose and to the groups in which the patients participated.

### 2.4. Statistical Analysis

For all outcome measures, unrelated sample Student's *t*-tests were employed for between group comparisons at the baseline. Multivariate analyses of variance (MANOVA) were employed, including group (Trained versus Control) and time (pretest versus posttest) as factors, with repeated measures on the second factor. Separate MANOVA analyses included clinical variables and gait variables, respectively. Univariate analyses were employed whenever MANOVA revealed interaction between the factors. Significant interactions were followed up with post hoc comparisons using Tukey honestly significant difference (HSD) procedure. The statistical analysis employed SPSS for Windows, with an alpha level of 0.05. 

## 3. Results

Five participants in the TG dropped out of the study: three due to the time commitment, one moved to another city, and one died. Thus, data from 19 individuals who completed the training protocol (10 women and 9 men) and 10 control participants (4 women and 6 men) were used for analysis. Although the participants' distribution procedure was not random, the two groups were not significantly different in demographic, clinical, and gait data at baseline. *P* values of *t*-tests are outlined in Tables 1, 3, and 4, respectively. 

The first MANOVA did not reveal interactions between factors (Wilks' Lambda = 0.846, *F*_(5,23)_ = 0.840, *P* = 0.535) for clinical variables. Clinical data from pre- and posttests are shown in Table 3. 

The second MANOVA revealed interactions between factors (Wilks' Lambda = 0.838, *F*_(7,79)_ = 2.184, *P* = 0.044) for gait variables. Univariate analysis for interaction between groups and time revealed significant differences for stride length (*F*_(1,85)_ = 8.205, *P* = 0.005), and stride velocity (*F*_(1,85)_ = 9.290, *P* = 0.003), and trend for stride duration (*F*_(1,85)_ = 3.608, *P* = 0.061). Tukey's HSD post hoc test revealed that only the TG group had a statistically significant improvement of these gait parameters at posttest when compared with pretest. Gait data for pre- and posttests are shown in Table 4.

## 4. Discussion

The aim of this pilot study was to identify the effects of a 6-month, multimodal exercise program on clinical and kinematic gait parameters in PD patients. Primarily, it is important to attest that the applied intervention had no adverse event, which indicates that it could be carried out by people with PD in a safe manner. The current study brings a new approach to the possibilities that physical exercise can offer for rehabilitation in PD.

The purposed multimodal exercise program has found improvement in some kinematic gait parameters, but not in clinical data, for the TG. Although promising, these results should be interpreted with caution, as the current study has some important methodological limitations: (i) selection bias in the group assignment; (ii) the lack of blinding of participants to group assignment; (iii) sample size. 

Regarding kinematic gait parameters, the proportional durations of stride phases and cadence did not change as an effect of the enrolment in the program. The main gait-related findings after participation in the program include improvements in stride length, stride duration, and stride velocity (7.7%, 8.5%, and 14%, resp.). The TG approached or reached the accepted means for Brazilians healthy elderly individuals—stride length: 117 cm; stride duration: 0.99 s; stride velocity: 120 cm/s [27]. Improvements in gait parameters have been shown to be of clinical relevance for elderly [28, 29], which would represent positive impact on quality of life. Improvement in usual gait speed after a follow-up of one year predicts a substantial reduction in mortality [28]. Also, Cesari et al. [29] have suggested that usual gait speed of less than 1 m/s identifies persons at high risk of health-related problems (persistent severe lower extremity limitation, death, and hospitalization) in well-functioning older people. With this regard, it is important to note that the participants of TG have obtained a stride velocity value higher than this cutoff point at posttest. 

In addition to current findings, our group has previously demonstrated a positive effect of the purposed program on executive functions [16] and on functional mobility and balance [17] in PD patients. Since multimodal exercise programs could have positive impact on motor and cognitive outcomes [16, 17], they should be further explored as a possible intervention tool for PD patients.

Both the clinical and gait parameters of PD patients tend to worsen progressively. Alves et al. [30] found similar mean annual declines in the UPDRS-III score and the H&Y staging, at 3.1% and 3.2%, respectively. Also, the UPDRS-II score declined 3.5% per year. Our findings do not point to a positive effect of purposed program over clinical data. It seems important to note that none of the clinical or gait parameters worsened for the CG in the study. It could be argued that six months does not represent enough time to observe significant declines in clinical or gait parameters of mild to moderate PD patients. Thus, it is an important aspect to guide future long-term exercise trials: periods longer than six months should be considered.

The rationale to choose a multimodal exercise intervention derived from the fact that the specificity of exercises is unclear relative to exercise trials in the rehabilitation literature for PD patients. Researchers have had difficulty reaching a consensus on which type of exercise is most beneficial for gait parameters. For example, body-weight supported exercises on a treadmill (three days a week for four weeks) resulted in significant gait and symptom improvements [11]. Walking on a treadmill at a speed greater than overground walking speed (three times per week for eight weeks) was also efficient in ameliorating gait parameters such as walking speed, stride length, and cadence [13]. Nieuwboer et al. [14] employed visual and auditory cues, as well as verbal instructions (three times per week for six weeks), which appeared to help increase the stride length of PD patients. The use of resistive training for lower limbs and abdominal muscles (twice a week for eight weeks) reveals a significant increase in stride length and velocity [15]. Sage and Almeida [4, 5] demonstrated that sensory, attention-focused exercise (three days a week for 12 weeks) can benefit the symptoms and gaits of PD patients. In this context, we can speculate that specificity of exercise is not a critical factor in improving gait parameters.

All of these exercise trials were designed specifically to improve gait parameters and had short-term durations (between 4 and 12 weeks). However, we suggest that PD patients should be enrolled in exercise programs for long periods (at least for 24 weeks), and that such programs also should attempt to correct a range of impairments caused by PD, which ultimately could result in improvements for quality of life. Our findings suggest that this could be possible with a 6-month, multimodal exercise program. In addition, long-term exercise programs could provide another benefit: fighting against disease progression (protective effect of exercise). Over short periods, it is difficult to assess the effects of exercise related to the progression of PD. This is supported by the fact that exercise studies of an animal model of PD have demonstrated increased survival of nigrostriatal dopaminergic neurons, suggesting a potential protective effect of exercise as well [31]. Therefore, it may be argued that these benefits could be achieved by a physically active lifestyle.

It would be useful to design other studies to help improve the understanding of how exercise trials affect the gait parameters of PD patients. Future long-term exercise trials should include midpoint assessment to verify specific short- and long-term exercise-related changes on clinical and gait parameters. It could also be useful to review walking tasks that are utilized to evaluate gait. For example, walking on a treadmill or on a walkway is different than walking in domestic and community environments. Locomotion over uneven terrains (obstacle crossing task) [26], or under postural threat (constrained and elevated floor) [25], is a more complex tasks and should be explored relative to PD patients enrolled in exercise programs.

In conclusion, the purposed 6-month, multimodal exercise program has found improvement in some kinematic gait parameters of mild-to-moderate idiopathic PD patients. Further study in the form of a randomized controlled trial would be required to establish effectiveness of our program. The current findings should be useful in guiding the design of such studies.

## Figures and Tables

**Table 1:** Characteristics of participants.

	TG (*n* = 19)		CG (*n* = 10)		*P* value
	Mean (sd)	Range	Mean (sd)	Range	*t*-test
Age (years)	67.5 (8.3)	52–81	71.3 (8.1)	55–84	0.242
Body height (cm)	159.4 (8.9)	142.8–176.5	160.8 (9.1)	147.1–173.7	0.694
Body mass (kg)	68.9 (13.5)	46.5–91.6	63.5 (9.8)	46–75	0.268
Years since diagnosis	3.8 (3.9)	1–19	4.4 (2.8)	1–10	0.695

Legend: TG: Trained group; CG: Control Group.

**Table 2:** Designed phases of the 6-month, multimodal exercise program with progressive increments on load and complexity for people with Parkinson's disease (adapted from Tanaka et al. [16]).

Phases	Capacities		
	Coordination	Muscular Resistance	Balance
Phase 1	Upper and lower limbs movements.	Exercises without weights.	Recreational activities that stimulated the vestibular system.
Phase 2	Trunk movements were added to upper and lower limbs movements.	Light-weight equipment (hoops, ropes, and batons).	Recreational activities that stimulated the visual and vestibular systems.
Phase 3	Trunk movements were substituted by head movements.	Heavier equipments (barbells, ankle weights, medicine balls).	Recreational activities that stimulated the visual and somatossensorial systems.
Phase 4	Head, trunk, and upper and lower limb movements.	Load was again increased with heavier equipment for resistance training (increase of intensity) or increased repetitions (increased volume).	Recreational activities integrated the vestibular, visual, and somatossensorial systems.
Phase 5	Four different movement sequences, two of which were the same for upper and lower limbs and two other sequences that alternated movements for upper and lower limbs in place and in movement.	Exercises were done with weights: leg press, pulley, seated cable rows, peck deck, and bench press. Load was adjusted according to patients' convenience (in two series of 15 repetitions).	Recreational activities included static balance, dynamic balance, half-turn, and complete turn (all with visual cues).
Phase 6	Four sequences of different movements, two sequences of alternating movement for upper and lower limbs and two sequences of different movement for upper and lower limbs, with or without trunk movement and equipment (balloons, balls, hoops, and rope).	The same exercises with load increase. Series of 15 repetitions were added.	Recreational activities were composed of activities with tactile cues.

**Table 3:** Means and standard deviations for each clinical dependent variable at pre and posttest and *P*-value for the comparison at baseline.

Measure	Pretest		*P* value at baseline TG versus CG	Posttest		Interaction between factors
	TG (*n* = 19)	CG (*n* = 10)		TG (*n* = 19)	CG (*n* = 10)	
	Mean (sd)	Mean (sd)		Mean (sd)	Mean (sd)	
H&Y	1.5 (0.75)	1.6 (0.88)	0.688	1.6 (0.73)	1.7 (0.85)	ns
UPDRS-I	3.9 (2.7)	2.8 (1.4)	0.143	3.6 (2.4)	2.5 (1.9)	ns
UPDRS-II	12.2 (7.4)	12.2 (4.9)	0.997	11.2 (7.2)	12.5 (6.7)	ns
UPDRS-III	22 (12.7)	28.4 (9.5)	0.175	22.5 (14.4)	28.9 (9.1)	ns
MEMS	25.8 (4.1)	23.7 (5.5)	0.256	25.8 (4)	24.7 (4.5)	ns

Legend: TG: Trained group; CG: Control Group; H&Y: Hoehn and Yahr Rating Scale; UPDRS: Unified Parkinson's Disease Rating Scale; MEMS: Mini-Exam of Mental Status; ns: nonsignificant interaction between factors.

**Table 4:** Means and standard deviations for each gait dependent variable at pre and posttest and *P*-value for the comparison at baseline.

Measure	Pretest		*P* value at baseline TG versus CG	Posttest		Interaction between factors
	TG (*n* = 19)	CG (*n* = 10)		TG (*n* = 19)	CG (*n* = 10)	
	Mean (sd)	Mean (sd)		Mean (sd)	Mean (sd)	
Stride length (cm)	95.1 (14.8)	94.8 (20.7)	0.95	102.4 (15.5)	96.6 (25.7)	*TG: post > pre
						CG: post = pre
Stride duration (s)	1.06 (0.17)	1.07 (0.1)	0.683	0.97 (0.11)	1.03 (0.1)	trend
						TG: post < pre
						CG: post = pre
Stride velocity (cm/s)	92.7 (21.1)	90.2 (24.9)	0.625	105.7 (15.5)	94.8 (28.8)	*TG: post > pre
						CG: post = pre
Cadence (strides/s)	0.97 (0.13)	0.94 (0.09)	0.387	1.04 (0.12)	0.98 (0.09)	ns
Swing phase (%)	36.5 (4.1)	36.3 (2.4)	0.771	37.2 (2.3)	36.9 (2.6)	ns
Single support (%)	37.5 (3.7)	37.6 (3.4)	0.906	38.3 (2.3)	38.3 (2.7)	ns
Double support (%)	26 (7.3)	26.1 (4.9)	0.924	24.5 (4)	24.8 (4.5)	ns

Legend: TG: Trained group; CG: Control Group; *:significant interaction between factors (*P* < 0.05); ns: nonsignificant.
